# Ecological Factors Influencing Nest‐Site Selection and Colony Size in Village Weavers *Ploceus cucullatus*

**DOI:** 10.1002/ece3.72840

**Published:** 2026-01-08

**Authors:** Joseph Izang Ibrahim, Ojodomo Godday Simon, Emmanuel Elisha Barde, Jacinta Abalaka, Shiiwua A. Manu

**Affiliations:** ^1^ School of Biodiversity, One Health and Veterinary Medicine University of Glasgow Glasgow UK; ^2^ A. P. Leventis Ornithological Research Institute University of Jos Centre of Excellence in Ornithological Training and Research Laminga Nigeria; ^3^ Department of Biological Sciences, FitzPatrick Institute of African Ornithology University of Cape Town Cape Town South Africa; ^4^ African Nature Investors (ANI) Foundation Gasha Gumti National Park Serti Nigeria; ^5^ Department of Zoology, Faculty of Natural Sciences University of Jos Jos Nigeria

**Keywords:** breeding population size, group breeding, Jos Plateau, nesting aggregation, Savannah birds, sub‐Saharan Africa

## Abstract

Understanding the ecological factors influencing breeding site selection in animals could offer valuable insights into how species navigate the trade‐offs between predator avoidance, resource availability, habitat requirements and human influence. In this study, we investigated the factors influencing the choice of nesting site and colony size in Village Weavers ( *Ploceus cucullatus* ) between wild habitats and human settlements on the Jos Plateau, Nigeria. We collected data from 41 colonies to examine how variables such as nesting substrate, nesting tree height, nesting tree size (girth), tree density around the colony, percent canopy cover, distance to accessible water, and habitat influence colony size. Our results reveal a divergence in nest‐site preferences across habitats. Colonies in wild environments had a higher preference for larger, taller trees and more densely vegetated areas, while colonies in human settlements were more strongly associated with proximity to water sources, reflecting the influence of nearby seasonal farms rich in grains and vegetables. These findings show a context‐dependent nesting strategy in Village Weavers, with wild colonies favoring sites that offer structural protection, while colonies around human settlements prioritizing access to food sources. The study shows how human‐modified landscapes can reshape nesting preferences in adaptable bird species and shows the fine‐scale ecological factors that influence nesting decisions in synanthropic birds across land‐use gradient in sub‐Saharan Africa.

## Graphical abstract

This study investigates how ecological and human‐related factors influence nest‐site selection and colony size in Village Weavers ( *Ploceus cucullatus* ) on the Jos Plateau, Nigeria. Findings reveal habitat‐specific nesting strategies, with wild colonies favoring structural protection and settlement colonies prioritizing proximity to food source.

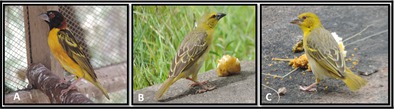

## 1 Introduction

Nest‐building is an essential evolutionary activity shared across many animal taxa, an act that is as important as it is diverse. Nest structure, and the choice of nest‐site, varies considerably between and within taxa, but serves the primary purpose of providing shelter, safeguarding offspring, and supporting reproductive success (Hansell 2000). In birds, for example, some hypotheses have been postulated to explain the mechanisms underlying nest‐site selection in relation to predation risk mitigation. The nest‐concealment (Martin 1993), potential prey‐site (Martin and Roper 1988; Chalfound and Martin 2009), and predator‐barrier hypotheses offer some explanation on how nest‐site selection could reduce predation risk and enhance the survival of both offspring and the parents. In addition to the construction of nests, the company a bird keeps can significantly influence its breeding success and survival (Momberg et al. 2022; Lee et al. 2024). Consequently, many species have evolved different anti‐predator strategies to enhance survival, including communal roosting, flocking in groups, colonial breeding, and nesting near human settlements (Madin et al. 2015; Lee et al. 2024). These colonies, whether formed temporarily for breeding or more permanently for roosting, confer a range of ecological benefits: enhanced vigilance against predators (Cresswell 1997; Latif et al. 2011), social facilitation in mate finding and care of young (Velando and Márquez 2002), and in some species, the capacity for rapid replacement of lost mates or nests (Ventura et al. 2021). Therefore, the choice of a nest‐site is one of the most crucial life‐history decisions a bird makes. It affects not only the likelihood of survival for the offspring but also the cost of reproduction for the parents (Kellett et al. 2003; Haynes et al. 2014; Pev et al. 2021).

Village Weavers, native to sub‐Saharan Africa, offer a compelling model for studying nest‐site selection due to their ecological flexibility. They are highly gregarious, colonial, and synanthropic, often nesting in large groups near human settlements and agricultural landscapes (Simon et al. 2023; Omotoriogun et al. 2025). Their woven nests are conspicuous, suspended from trees, often situated near roads, farms, rivers, or residential areas (Craig and de Juana 2020). However, Village Weavers are also known to nest in the wild, away from human disturbance (Borrow and Demey 2020). The wild colonies may have a preference for different habitat conditions to those in human settlements in terms of vegetation structure around the nest‐site, suitable nesting substrate, and characteristics of nesting substrate. This raises important questions about whether habitat characteristics for nesting differ between wild and human‐associated colonies, and how these differences influence colony size.

In this study, we investigated how colony size varies with nesting substrate characteristics and environmental context, comparing colonies in the wild habitats with those in human settlements. We hypothesized that wild colonies would prioritize nest‐site that offers structural characteristics that provide security from predation (such as larger trees and denser vegetation), whereas human‐associated colonies would prioritize proximity to food resources over structural security, as predation risk is reduced near human settlements. Furthermore, we expected larger colony sizes in human‐associated habitats due to lower predation pressure. Understanding these could offer insight into how species balance the often‐conflicting demands of safety, resource availability, and social dynamics.

## 2 Methods

### 2.1 Study Site

**FIGURE 1:**
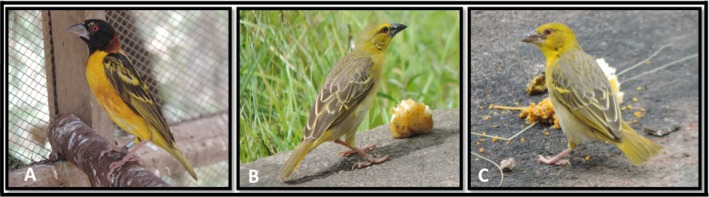
Plumage variation in the Village Weavers ( *Ploceus cucullatus* ). (A) Male in breeding plumage, (B) Male in nonbreeding plumage, and (C) Female.

This study was conducted in Jos East Local Government Area in the Middlebelt region of Nigeria. The Local Government Area lies within longitude 08°52′ E to 09°07′ E and latitude 09°14′ N to 10°01′ N with a total land area of about 1020 km^2^, situated approximately 10 km Northeast of Jos, the Plateau State capital. The region comprises of high plains about 1300 m above sea level and some granite hill ranges that rise to 1700 m above sea level (Figure 1; Usieta et al. 2013). Guinea savanna is the general vegetation pattern consisting of predominantly tall grasses, shrubs, lianas, and spatially distributed tall trees (Joel et al. 2024; Matthew et al. 2024). Within the guinea savannah are many different micro‐habitat types including rocky outcrops, gallery and riparian forests, farmlands, open savannahs, woodlands, and wetlands. Due to the heterogeneous habitat and complexity in the landscape, it strongly supports biodiversity and serves as home to various bird, mammal, reptile, amphibian, and invertebrate species (Joel et al. 2024). For example, Amurum Forest Reserve (112 ha woodland fragment) alone harbors over 300 bird species (1 endemic), 150 butterfly species (2 endemics) and many plants of both conservation and cultural significance.

### 2.2 Data Collection

We study breeding colonies of Village Weavers over a period of 3 months (August to October 2017). Village Weavers are gregarious, dimorphic, and polygamous passerines that are native to sub‐Saharan Africa (Borrow and Demey 2020). Adult male Village Weavers in breeding plumage have a black crown, face, chin, and throat, dark chestnut nape, and neck sides, mottled black and yellowish back forming a V‐shape, and a yellow rump with an olive tail (Figure 1A), while males in nonbreeding plumage have an olive‐gray‐green crown, gray‐brown back, and dark streaks on central feathers, a black bill, and yellowish bellied (Figure 1B). Females have an olive‐gray‐green crown, gray‐brown back, dark streaks on central feathers, a pale bill, and white bellied (Figure 1C) (Borrow and Demey 2020).

To ensure wide coverage of the study area and facilitate ease of movement during fieldwork, we systematically spread our sampling effort across the six smallest political divisions (districts) within Jos East Local Government Area. At least seven locations were randomly selected from each district, and colonies of Village Weavers were systematically searched for.

**FIGURE 2:**
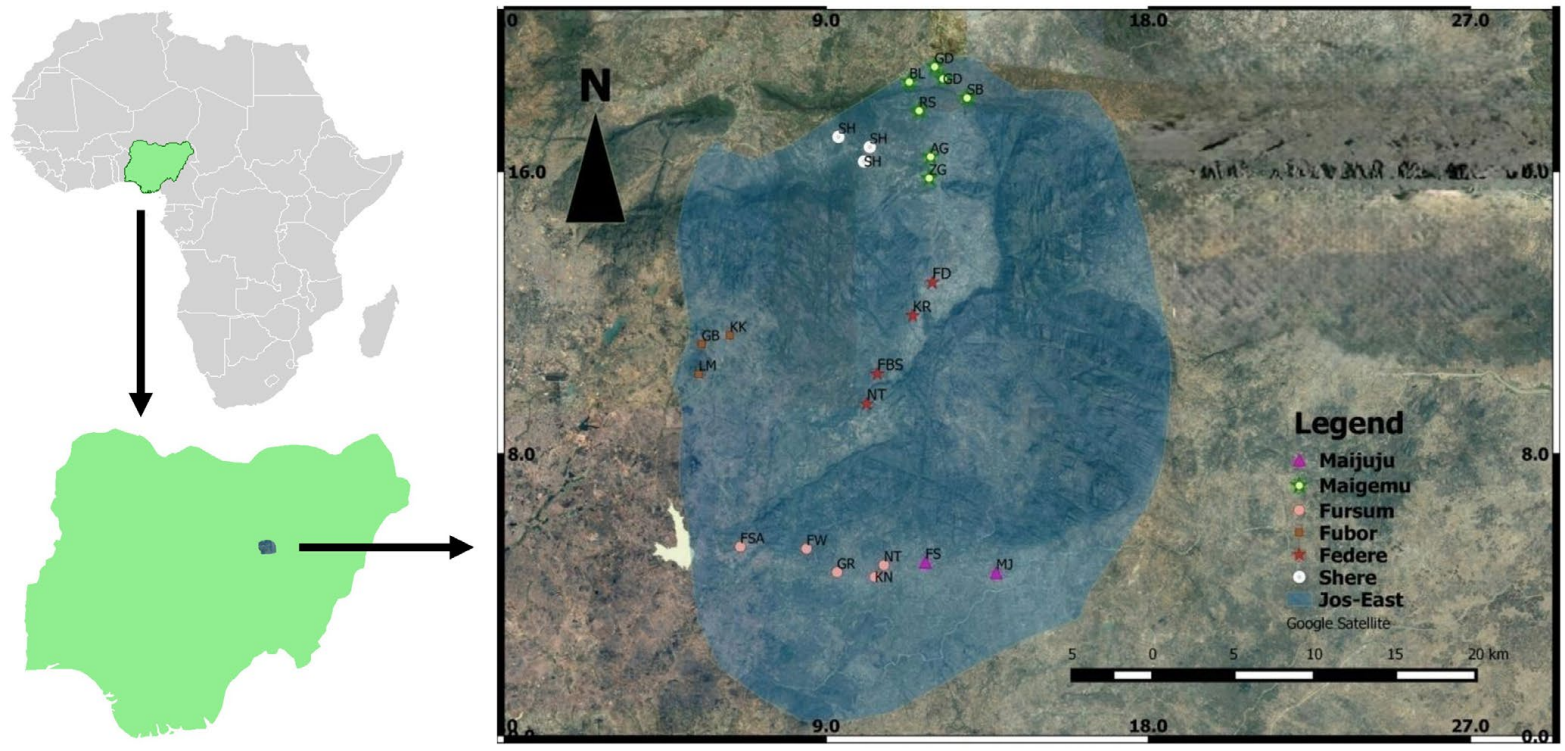
Map of the study area and sampling locations. Top left: Map of Africa showing Nigeria. Bottom left: Map of Nigeria showing the location of Jos East Local Government Area. Right panel: Satellite image showing the spatial distribution of Village Weavers colony sampling points across six districts in Jos East. Nest‐sites are color‐coded by district (where GD is Gada, SB is Shibiri, BL is Balo, RS is Rafin Sanyi, SH is Shere, AG is Angware, ZG is Zangam, FD is Federe, KR is Kurumin, FBS is Febas, NT is Nabur, FSA is Fusa, FW is Fewit, GR is Gora, KN is Kona, FS is Tere and MJ is Maijuju).

We marked the location of each colony using Gramin GPS65 device (Figure 2 right), and quantified habitat characteristics on three different scales. The first (microhabitat) included the plant substrate that harbored the nests and the colony location (human settlement vs. wild, hereafter habitat). Nest‐sites located within approximately 250 m of human habitation were classified as being in human settlements, as this distance represents the zone of frequent human presence, high noise, and disturbance. Although certain activities such as farming, livestock movement, and firewood collection may occur farther away, areas beyond this threshold experience minimal human traffic and transition into more natural vegetation and were thus classified as wild. At this level, we also identified the nesting plant species, measured its circumference at breast height (CBH), percentage canopy cover, estimated average nest height, and the height of the nesting tree. The CBH of each nesting tree was measured using a tape rule at a height of 1.3 m from the ground level (Manu et al. 2005). For trees with multiple branches at that height, the CBH of each of the branches was measured and the average was computed. The percentage canopy cover was estimated using the wrong side of a pair of binoculars (Manu et al. 2007), while the nesting tree height and average nest height were estimated by eyes, by the same observer throughout the study. The second scale included the vegetation patch around the nest‐site (mesohabitat) and was measured by counting the number of trees within a 100 m radius of each colony. In addition to vegetation parameters around each nest‐site, the distance of the nest‐site to the closest accessible water (open water body such as rivers, streams, lakes, and dams) was measured, and this constituted the third scale (metahabitat).

In this study, colony size was defined as the number of nests in each nest‐site on a single tree (hereafter, colony size).

### 2.3 Data Analysis

We fitted a generalized linear mixed model (GLMM) in the package glmmTMB version 1.1.11 (Brooks et al. 2017), assuming a truncated negative binomial distribution (family: truncated_nbinom2) to account for overdispersion and the absence of zero counts in our data, given that colony sizes cannot be zero. Our global model included the tree species that Village Weavers nests were found on as a fixed effect, along with other environmental covariates with all possible two‐way interaction with habitat (human settlement vs. wild). These covariates were: distance to the nearest water source, circumference at breast height of the nesting tree, percent canopy cover, nesting tree height, average nest height, and the number of trees within a radius of 100 m to the colony. We used site as a random intercept to account for potential clustered data within site, since multiple colonies were sampled within the same site.

Model selection was done using stepwise elimination (both forward and backward) based on Akaike's Information Criteriaon (AIC) to identify the most parsimonious model, balancing explanatory power and model complexity (Bozdogan 1987), using the *step* function (R core Team 2025). Although information‐theoretic and model‐averaging approaches are increasingly advocated (e.g., Guthery et al. 2003; Genell et al. 2010), stepwise AIC selection was adopted here as a pragmatic and interpretable approach suited to our objectives. We then performed model diagnostics using the package DHARMa version 0.4.7 (Hartig 2024) by simulating residuals and checking for deviations from assumptions such as overdispersion and residual patterns. Multicollinearity among predictors was assessed using the function *check_collinearity* in the package performance version 0.14.0 (Lüdecke et al. 2021), ensuring variance inflation factors (VIFs) remained within acceptable ranges (commonly < 10), indicating low collinearity except for interactions. Our final model included the following variables: distance to water, colony location (i.e., whether the colony was in the wild or within human settlement), circumference at breast height of the nesting tree, nesting tree height, and number of trees within the colony site. Additionally, the model retained the interactions between distance to water and colony location, nesting tree height and colony location, as well as between number of trees and colony location. We used the *tab_model* function in the package sjPlot version 2.4.17 (Lüdecke 2024) to compute the effects of the final model, suppressing variance components such as the intraclass correlation coefficient (ICC) while including the log‐likelihood and AIC values for model assessment. Our final model explained a substantial proportion of the variance in colony size (marginal *R* ^2^ = 0.71).

Furthermore, we performed a Principal Component Analysis (PCA) to explore patterns in the environmental and structural characteristics of Village Weavers colonies. Prior to PCA, we scaled all continuous predictor variables (e.g., nesting tree height, diameter at breast height of nesting tree, canopy cover, number of trees, and distance to water) to unit variance. Additional useful information such as habitat (human settlement or wild) and colony size were not included in the PCA computation but were incorporated in the visualization phase to interpret within and between groups patterns. We performed the PCA using the *prcomp* function (R Core Team 2025), which performs a singular value decomposition on the scaled data matrix. For visualization, we used *the fviz_pca_biplot* function from the package factoextra version 1.0.7 (Kassambara and Mundt 2020). We used Microsoft PowerPoint (Microsoft Corporation 2025) to rearrange our figures.

All data exploration and analyses were carried out in R version 4.5.0 (R Core Team 2025), and plots were prepared using ggplot2 version 4.0.0 (Wickham 2016).

## 3 Results

**FIGURE 3:**
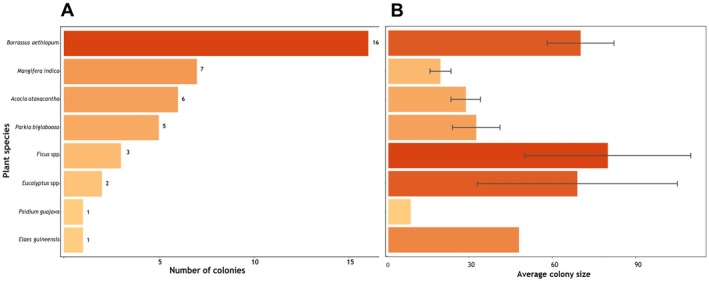
Colony distribution and sizes across plant species. (A) Number of colonies found on each plant species. (B) Average colony size per plant species.

**FIGURE 4:**
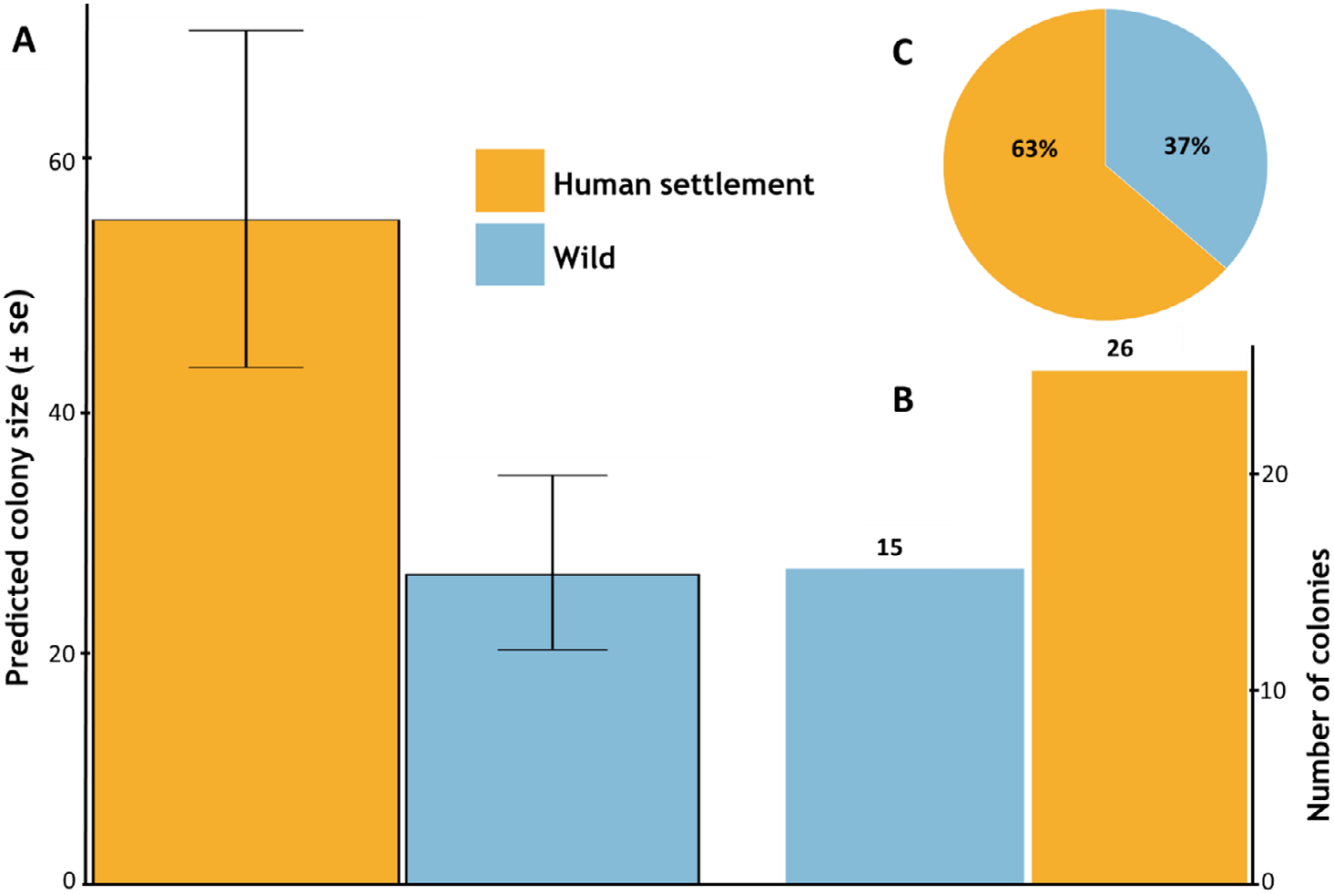
Differences in (A) predicted mean colony size, (B) number of colonies, and (C) proportion of colonies between habitat types (human settlements versus wild).

We recorded a total of 41 Village Weavers colonies in 21 sites, covering an approximate area of 900 km^2^ (Figure 2 right). The minimum colony size recorded was 2 while the maximum was 175. At the microhabitat level, we found that Village Weavers nest on a wide variety of plant species, ranging from native trees of ecological and economic importance, such as *Parkia biglobosa* and *Borassus aethiopum* , to exotic and invasive species like *Eucalyptus* spp. Colonies were observed on both individual tall trees like *Borassus aethiopum and Eucalptus* spp, and in dense thickets, particularly those dominated by *Acacia ataxacantha* and grasses. We found the highest number of colonies on *Borassus aethiopum* , while *Psidium guajava* and *Elaeis guineensis* had the lowest number of colonies (Figure 3A). In terms of colony size, Ficus species had the largest average colony size while *Psidium guajava* had the least (Figure 3B). Colonies were common around settlements with agricultural lands especially grains such as *Sorghum* spp., *Pennisetum* spp., and *Zea maize*. Nest location varied considerably. We recorded many colonies near human settlements, and some were found kilometers into the wild. Colony size significantly differed by habitat (Table 1), with colonies in human settlements having significantly larger sizes compared to those in the wild (Figure 4A). In terms of absolute numbers, we similarly found more colonies in human settlements compared to wild (Figure 4B), accounting for 63% of the total number of colonies found in this study (Figure 4C).

**TABLE 1:** Mixed effects truncated negative binomial model of Village Weavers colony size as a function of environmental variables. Site was modeled as a random intercept.

Predictors	Parameter estimate	Colony size (truncated NB model)		
		Incidence rate ratios	CI	*p*
Intercept	3.52	33.87	8.06–142.36	**6.78e‐07**
Distance to water	−0.01	0.99	0.99–1.00	**0.0013**
Colony location [Wild]	−3.75	0.02	0.00–0.16	**9.97e‐05**
Circumference at breast height	0.41	1.51	1.12–2.04	**0.007**
Nesting tree height	0.02	1.02	0.85–1.23	0.796
Number of trees	0.01	1.01	0.98–1.04	0.712
Distance to water: Colony location [Wild]	0.01	1.01	1.00–1.01	**0.000237**
Colony location [Wild]: nesting tree height	0.25	1.29	1.03–1.61	**0.029**
Colony location [Wild]: number of trees	0.11	1.11	1.01–1.22	**0.036**
**Random effects**				
*σ* ^2^		0.22		
*τ*_00 site_		0.00		
*N*_site_		41		
Observations		41		
Marginal *R* ^2^		0.71		
Log‐Likelihood		−177.72		

*Note:* Bold values indicate statistical significance at *p* < 0.005.

**FIGURE 5:**
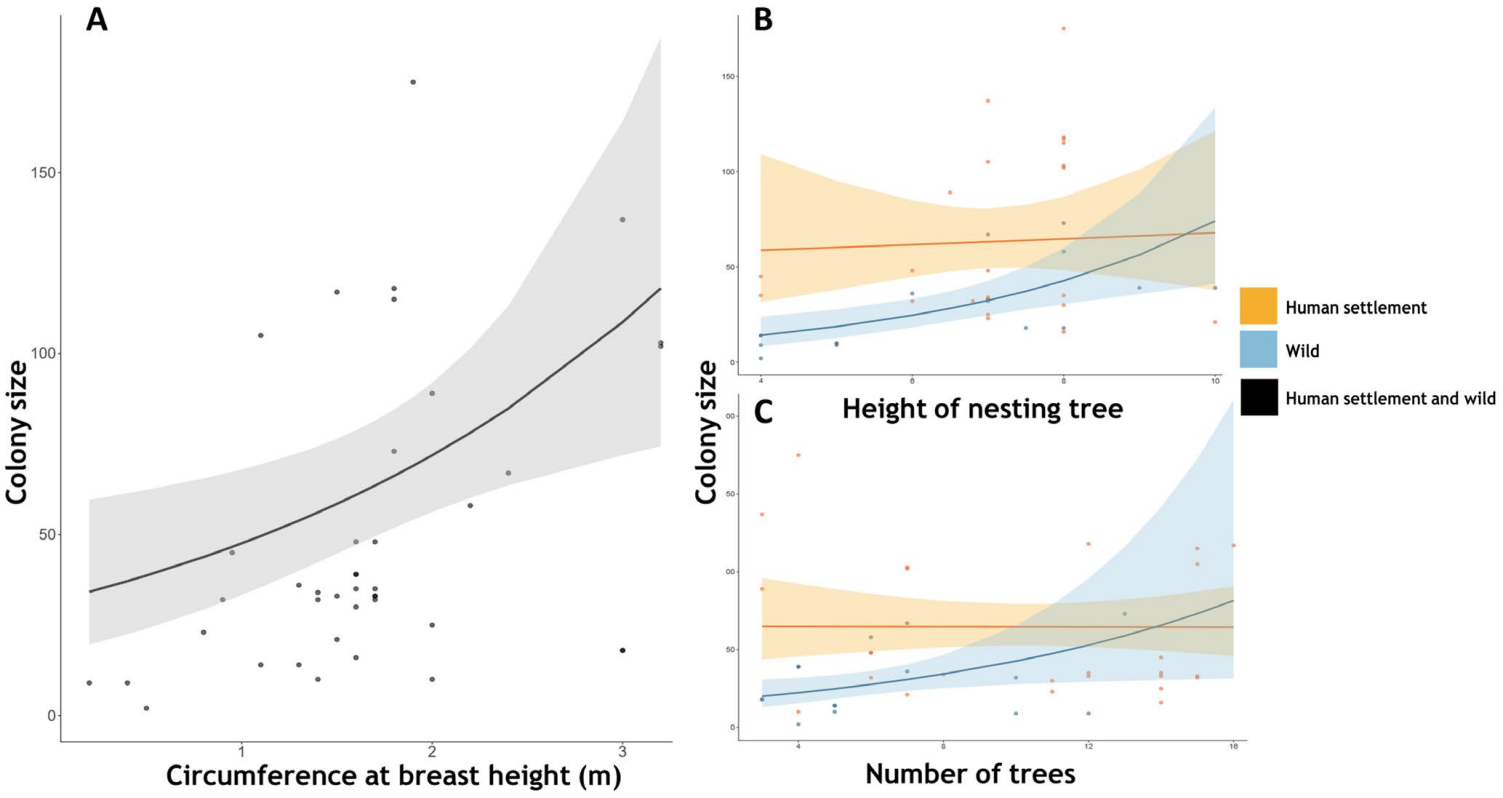
Effect of (A) circumference at breast height, (B) nesting tree height, and (C) number of trees within 100 m radius of nest‐site on colony size.

At the mesohabitat level, CBH, nesting tree height, and number of trees around the colony were important predictors of colony size. CBH (indicator of tree size) had a significant effect on colony size (Table 1) and was positively correlated (Figure 5A). Colonies in the wild generally have higher preferences for both taller trees and higher tree densities (number of trees), this is shown by larger colony size with taller and bigger trees, those in human settlement are not influence by either tree height (Figure 4B) or tree density (number of trees around the nest‐site) (Figure 5C).

**FIGURE 6:**
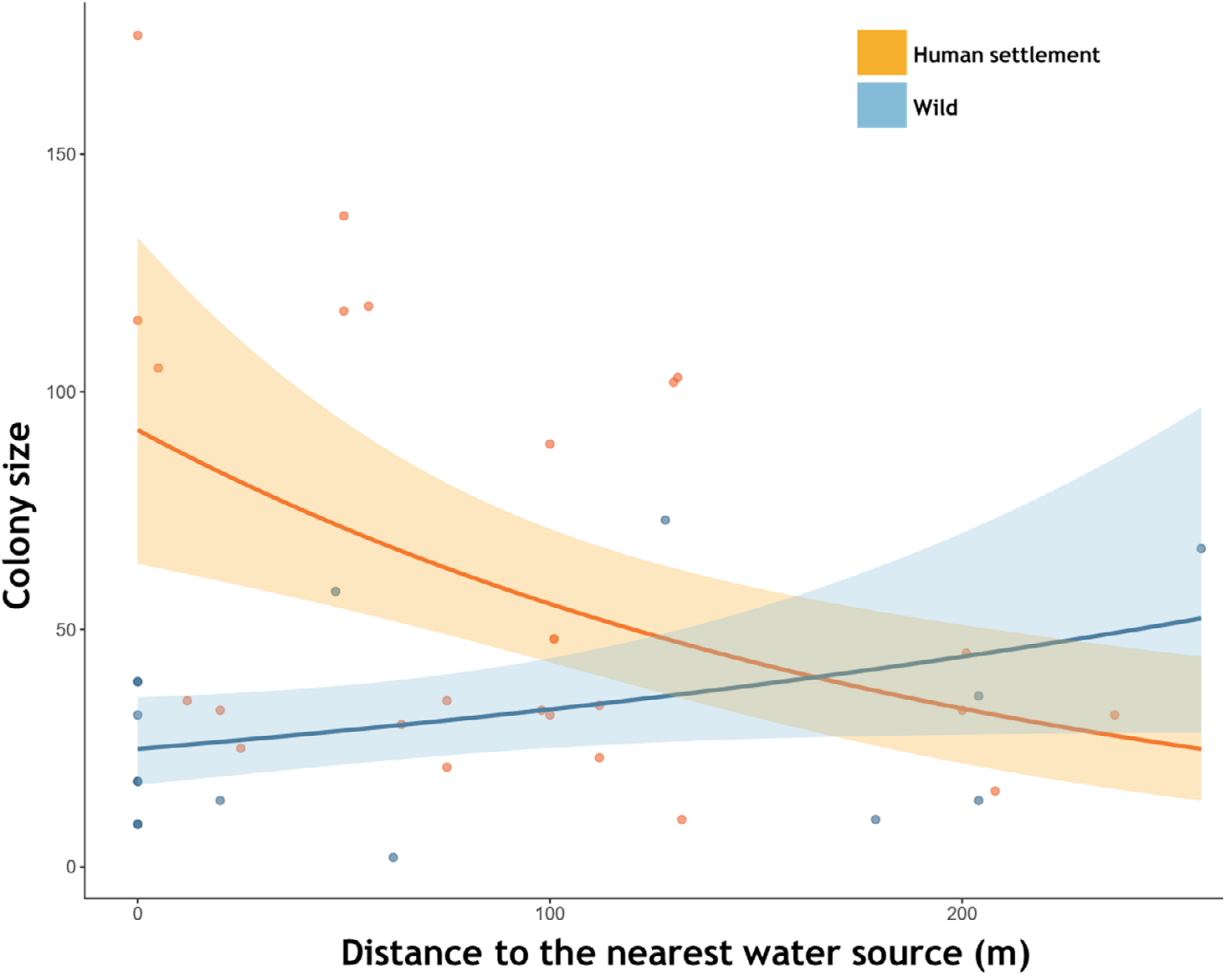
Effect of distance to nearest water source on colony size in different habitats.

At the metahabitat level, distance to nearest water source influenced colony size significantly (Table 1), which also varied with habitat, indicating a complex relationship accentuated by habitat. In the wild, the colony had a positive relationship with distance to nearest water source (Figure 6), whereas in human settlements, there was a negative correlation with colony size (Figure 6).

**FIGURE 7:**
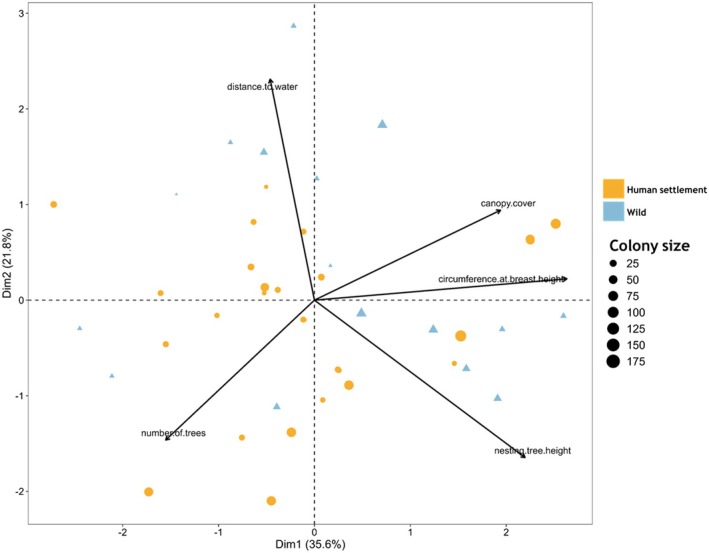
Principal Component Analysis (PCA) biplot of Village Weavers' colony distribution based on habitat characteristics and distance to accessible water across two habitat types (human settlements and the wild). Arrows represent the loadings of environmental variables, indicating their contribution to the ordination.

The first two principal components of our PCA explained a combined 57.4% of the total variance in the dataset (Figure 7; Table 2), with the first dimension (*x*‐axis) explaining 35.6% and the second dimension (*y*‐axis) explaining about 21.8% (Figure 7; Table 2). Colonies in human settlements and wild habitats show partial separation along the first dimension, suggesting structural differences between the habitats. Nesting tree height, canopy cover and circumference at breast height of nesting tree loaded positively on the first dimension and were more strongly associated with colonies in the wild habitat (Table 2), indicating that colonies in that habitat tended to occur on larger trees (Figure 7). Distance to water loaded strongly on the second dimension in the positive direction and is primarily associated with colonies in human settlement, implying a stronger association with proximity to water bodies in colonies in the habitat. The number of trees showed a negative association with the first dimension (Table 2), indicating that colonies in human settlement were often found in areas with more trees but smaller individual trees compared to those in the wild. Colony size varied widely across both habitats, with larger colonies clustered among human settlement sites (Figure 7).

**TABLE 2:** Environmental variable characteristics and their contributions to principal components. Descriptive statistics (range and mean ± standard deviation) summarize variable distributions, while loadings indicate strength and direction of relationships with principal components. Dim1 and Dim2 explain 35.6% and 21.8% of the total variance, respectively.

S/n	Variable	Range (min–max)	Mean	Dim1 (35.6%)	Dim2 (21.8%)
1	Distance to water (m)	0.00–258.00	85.32 ± 77.04	−0.14	0.72
2	Circumference at breast height (m)	0.20–3.20	1.67 ± 0.70	0.82	0.07
3	Percent canopy cover	20.00–90.00	57.2 ± 14.53	0.61	0.29
4	Number of trees	3.00–16.00	8.85 ± 4.34	0.69	−0.52
5	Nesting tree height (m)	4.00–10.00	6.82	−0.49	−0.46

## 4 Discussion

Our findings provide support for some ecological hypotheses concerning nest‐site selection by birds. Tree size (as measured by girth) was positively associated with colony size, consistent with the Nest‐Concealment Hypothesis, which hypothesize that larger trees provide better structural cover and concealment for nests, reducing the risk of detection by predators (Li and Martin 1991; Martin 1993). In wild habitats, colony size also showed a positive association with tree height and density. This pattern suggests that Village Weavers tend to establish colonies on substrate that confer reduced detectability and accessibility to predators. Large, tall trees provide both vertical separation from ground‐based threats, and denser vegetation cover makes it harder to access nests from visually hunting predators such as raptors. Our results also support the Predator‐Barrier Hypothesis, which emphasizes physical inaccessibility of nests as a mechanism to reduce predation (Caro 2005; Amo et al. 2017). In the wild, natural predators of many passerines (e.g., Village Weavers) such as monitor lizards, arboreal snakes, and birds of prey are more common than around human settlements (Chalfoun et al. 2002; Quinn and Ueta 2008), the benefits of nesting in structurally formidable trees and denser vegetated areas are magnified. Consequently, colonies located high above ground level or within dense vegetation may experience reduced predation risk, enhancing the survival prospects of both adults and offspring. Such preferences reflect evolved behavioral adaptations, where persistent exposure to predation pressure favored individuals selecting nest sites with better structural protection. In this sense, nesting tree structure constitutes an integral component of the species' anti‐predator strategy (Collias and Collias 1964). Even though colony size in human settlements had a positive relationship with tree size, there was a weaker correlation with microhabitat features. Instead, colony size showed a stronger negative association with distance to water, suggesting a shift toward functional optimisation under reduced predation pressure, aligning with the Potential Prey‐Site Hypothesis (Burger 1981). In human settlements, nesting patterns are more strongly influenced by proximity to resource‐rich areas than by structural protection. This finding is ecologically meaningful in the regional context where the study was conducted. Across the Afro‐tropical savannah landscapes of central Nigeria, seasonal farming is prevalent around open water bodies. The farms typically cultivate grains (e.g., maize, millet, rice, sorghum) and vegetables, all known to be part of the Village Weavers' diet (Craig and de Juana 2020; Simon et al. 2023). Consequently, water bodies in human‐modified areas do not merely serve as hydration sources or microclimate regulators; they function as predictable food patches, attracting Village Weavers that rely on both grain availability and associated insect abundance. Moreover, human presence often results in the suppression or displacement of natural predators, reducing the perceived need for concealment or nesting at high elevation. Under these conditions, colonies form on less structurally robust trees but situated near reliable food sources. This pattern reflects an adaptive adjustment in nesting strategy, where selection favors resource proximity under low predation risk.

Overall, our findings illustrate a degree of context‐dependent flexibility in nesting strategies of Village Weavers, shaped by local ecological pressures. This divergence illustrates behavioral plasticity that allows the species to exploit both natural and human modified habitats, a trait that might have contributed to their wide distribution and abundance in West African savannah (Craig and de Juana 2020; Borrow and Demey 2020).

## 5 Conclusion

Our findings show marked differences in ecological nesting strategies in Village Weavers depending on whether they are in wild or human‐dominated environments. Colonies in the wild prioritize security, nesting in larger, taller, and more densely vegetated areas that offer concealment and protection against natural predators. In contrast, colonies around human settlements appear to prioritize resource accessibility, particularly proximity to water‐linked foraging zones in farming areas. These patterns reflect a dynamic balance between risk and reward, shaped by the structure of the local environment and the behavioral adaptability of the species.

## Author Contributions

**Joseph Izang Ibrahim:** conceptualization (lead), data curation (lead), formal analysis (lead), funding acquisition (lead), investigation (lead), methodology (lead), project administration (equal), resources (lead), software (lead), supervision (equal), validation (lead), visualization (lead), writing – original draft (lead), writing – review and editing (lead). **Ojodomo Godday Simon:** conceptualization (supporting), data curation (supporting), formal analysis (equal), funding acquisition (supporting), investigation (supporting), methodology (supporting), project administration (supporting), resources (supporting), software (equal), supervision (supporting), validation (equal), visualization (supporting), writing – original draft (supporting), writing – review and editing (supporting). **Emmanuel Elisha Barde:** conceptualization (supporting), data curation (supporting), formal analysis (supporting), funding acquisition (supporting), investigation (supporting), methodology (supporting), project administration (supporting), resources (supporting), software (equal), supervision (supporting), validation (supporting), visualization (supporting), writing – original draft (supporting), writing – review and editing (equal). **Jacinta Abalaka:** conceptualization (equal), data curation (supporting), formal analysis (supporting), funding acquisition (supporting), investigation (supporting), methodology (supporting), project administration (supporting), resources (supporting), software (supporting), supervision (equal), validation (equal), visualization (supporting), writing – original draft (supporting), writing – review and editing (equal). **Shiiwua A. Manu:** conceptualization (lead), data curation (equal), formal analysis (equal), funding acquisition (lead), investigation (equal), methodology (lead), project administration (lead), resources (lead), software (supporting), supervision (lead), validation (lead), visualization (supporting), writing – original draft (supporting), writing – review and editing (lead).

## Conflicts of Interest

The authors declare no conflicts of interest.

## Data Availability

All data supporting the findings of this study are publicly available in the Dryad Digital Repository at https://doi.org/10.5061/dryad.dv41ns2c0.
